# 2296: Functional analysis of the cutaneous microbiome in psoriatic disease

**DOI:** 10.1017/cts.2017.68

**Published:** 2018-05-10

**Authors:** Di Yan, Hsin-Wen Chang, Rasnik Singh, Kevin Lai, Kristina Lee, Ladan Afifi, Xueyan Lu, Derya Ucmak, Susan Lynch

**Affiliations:** University of California, San Francisco, CA, USA

## Abstract

OBJECTIVES/SPECIFIC AIMS: Psoriasis is one of the most common inflammatory diseases of the skin, affecting about 2%–3% of the US population. Despite its high prevalence, its pathogenesis remains poorly understood. The ability of the microbiome to modify host immunity and metabolism suggests that it may contribute to the development of psoriasis and its cardiometabolic comorbidities. This study aims to characterize the psoriatic skin microbiome and understand the functional role that these bacteria may play. METHODS/STUDY POPULATION: 16s rRNA sequencing of site-matched skin swabs from 8 psoriasis patients and 8 healthy controls was used to identify bacteria and determine their relative abundance and microbial community diversity in the sample. PICRUSt was used to infer the functional roles of the bacteria from 16s rRNA amplicon data. RESULTS/ANTICIPATED RESULTS: Lesional psoriasis skin had lower α diversity (*p*=0.04), less Actinobacteria (*p*=0.0001), but higher Firmicutes (*p*=0.009) compared with controls. At the genus level, lesional skin had more Alloiococcus (*p*=0.01) and Aerococcus (*p*=0.01) and demonstrated a trend towards lower Propionibacterium (*p*=0.08) and higher Gallicola (*p*=0.09) compared to controls. Interestingly, Alloiococcus (*p*=0.003) and Gallicola (*p*=0.04) were also higher in nonlesional skin compared with controls. Furthermore, lesional and nonlesional skin shared an increased abundance of *Acinetobacter* sp., *Staphylococcus pettenkoferi*, and *Streptococcus* sp., relative to controls. Lesional and nonlesional psoriasis skin did not differ significantly in microbiome composition. Predictive functional analysis revealed that both the healthy and psoriatic skin microbiome were enriched with bacteria capable of amino acid and carbohydrate metabolism suggest these functions might have a general role in host-microbe interaction. DISCUSSION/SIGNIFICANCE OF IMPACT: These data reveal intriguing differences in the cutaneous microbiome of psoriatic individuals and healthy controls and suggest that bacterial metabolism may play an important role in host-microbe interaction.

